# Profilometric Quantification of Wear-Track Degradation in FFF Kevlar-Reinforced ASA Composites

**DOI:** 10.3390/ma19102135

**Published:** 2026-05-19

**Authors:** Patricia Isabela Brăileanu, Marius-Teodor Mocanu, Nicoleta Elisabeta Pascu

**Affiliations:** 1 Department of Robotics and Manufacturing Systems, Faculty of Industrial Engineering and Robotics, National University of Science and Technology POLITEHNICA BUCHAREST, RO-060042 Bucharest, Romania; nicoleta.pascu@upb.ro; 2 National Institute for Laser, Plasma and Radiation Physics, RO-077125 Măgurele, Romania; 3 Doctoral School of Industrial Engineering and Robotics, National University of Science and Technology POLITEHNICA BUCHAREST, RO-060042 Bucharest, Romania

**Keywords:** additive manufacturing, fused filament fabrication, Kevlar-reinforced polymer, tribology, wear track profilometry, surface profilometry, aramid composites

## Abstract

Fused filament fabrication (FFF) produces components with characteristic topographical features that influence their tribological behavior. Because conventional roughness parameters may not fully describe the localized surface degradation of reinforced FFF polymers, this study evaluates the wear-track evolution of FFF aramid fiber-reinforced Acrylonitrile Styrene Acrylate (ASA) composites using a comparative profilometric framework based on pre-wear and post-wear measurements. Specimens with different infill configurations underwent dry sliding Ball-on-Disc tribological testing, followed by profilometric wear-track analysis and optical microscopy inspection. The macroscopic wear response exhibited a non-monotonic dependence on infill configuration. Under the present experimental conditions, the 30% infill configuration showed the most favorable average wear response, with the lowest wear volume and specific wear rate, whereas the 90% infill configuration showed the highest material loss. To compare the surface modifications induced by sliding, three derived relative profilometric descriptors were evaluated: Surface Texture Alteration Index (STAI), Peak Deformation Index (PDI) and Material Ratio Preservation Index (MRPI). These descriptors were used as complementary comparative parameters rather than replacements for standardized roughness or Abbott–Firestone-based measurements. Statistical analysis showed a very strong association between maximum wear-track depth and calculated volumetric material loss, indicating that deeper wear-track profiles were consistently associated with higher material removal within the investigated dataset. Furthermore, correlation analysis suggested that the initial material ratio may be more closely associated with the subsequent wear response than the initial arithmetic mean roughness. This study indicates that combining wear volume, wear-track geometry, optical microscopy and relative profilometric descriptors provides a useful comparative approach for evaluating degradation in FFF Kevlar-reinforced ASA components under sliding conditions.

## 1. Introduction

Additive manufacturing (AM), specifically fused filament fabrication (FFF), has restructured the fabrication of complex thermoplastic components [1,2,3]. Operating through the layer-by-layer extrusion of molten polymers, FFF is now capable of producing characteristic layered textures, stair-step surface roughness and anisotropic microstructures [4,5,6]. These particular morphological features, including inter-filament voids and characteristic surface ridges and valleys, can govern the mechanical and tribological behavior of the printed parts [7,8,9]. Hence, the surface topography of an FFF component acts as both the initial condition for contact mechanics and a primary source of third-body particles during sliding wear [1,10,11,12].

To enhance tribological and mechanical performance, filaments are frequently reinforced with high-performance fillers such as carbon fibers (CFs) and Kevlar [13,14]. Some integration of CF into PLA (PLA-CF) improves stiffness and reduces the specific wear rate. However, exposed or detached fibers can act as hard third-body abrasives, occasionally accelerating abrasive wear on both the polymer matrix and the counter-surface [1,9,15,16]. Kevlar is an aramid fiber known for its good toughness and impact resistance, that significantly enhances the structural integrity and wear resistance of polymer composites [9,12,17]. While Kevlar reinforcement improves load-bearing capacity, it can also generate deep wear profiles, significant debris and abrasive scratching under sliding conditions [9].

To understand the wear mechanisms of FFF polymer surfaces, researchers frequently employ profilometric techniques and scanning electron microscopy to capture detailed topographic maps of worn surfaces [6,18,19]. Surface characterization typically relies on standard amplitude parameters, such as the arithmetic mean deviation (*R_a_*), root mean square deviation (*R_q_*) and maximum profile height (*R_z_*) [9,12]. Moreover, the Abbott–Firestone curve is a significant analytical tool used to assess material ratios and surface characteristics, providing insights into load-bearing capabilities and the depth distribution of the printed profile [12].

Despite their widespread use in tribological surface characterization, conventional amplitude roughness parameters such as *R_a_*, *R_q_* and *R_z_* mainly provide scalar information regarding the vertical distribution of surface asperities and may not fully describe the complex degradation mechanisms of FFF-generated surfaces [20]. In AM polymer composites, especially those reinforced with fibers such as Kevlar, sliding wear can induce anisotropic topographical transformations including localized ridge collapse, fiber pull-out, debris accumulation and directional ploughing [15,21,22]. Under such conditions, conventional roughness parameters remain valuable for describing general roughness evolution. However, they may not fully capture the progressive modification of load-bearing morphology and surface texture distribution. Previous studies have similarly reported that FFF surfaces exhibit heterogeneous wear patterns, directional surface features and localized deformation phenomena that are not always adequately represented by single amplitude-based descriptors alone [23,24,25,26]. Therefore, additional profilometric descriptors and comparative surface analysis may provide complementary insight into the evolution of tribologically degraded FFF structures.

The current literature on FFF tribology predominantly focuses on macro-scale performance indicators, such as the coefficient of friction (*μ*), mass loss and overall wear volume [9,14,19]. Although these parameters are essential for describing the global tribological response, they provide limited information regarding the localized evolution of surface morphology during sliding wear. Similarly, Abbott–Firestone-based parameters, such as the material ratio, describe specific load-bearing characteristics of the surface profile, but do not directly express the relative transformation of the surface from the pre-wear to the post-wear state. This limitation is particularly relevant for FFF Kevlar-reinforced ASA composites, where surface degradation may involve simultaneous changes in roughness amplitude, peak morphology, material ratio, fiber pull-out, debris accumulation and localized modification of the load-bearing surface fraction [15,20,21,22,23,24,25,26]. Therefore, while conventional roughness and Abbott–Firestone parameters remain necessary baseline descriptors, their isolated interpretation may not be sufficient to compare the coupled topographical changes induced by sliding wear across different infill architectures.

To address this specific limitation, the present study evaluates the wear-track degradation of FFF Kevlar-reinforced ASA composites using a comparative profilometric approach based on conventional surface parameters measured before and after tribological testing. Three derived relative descriptors, calculated from standardized profilometric parameters, are considered: the Surface Texture Alteration Index (STAI), Peak Deformation Index (PDI) and Material Ratio Preservation Index (MRPI). These descriptors are not intended to replace standardized roughness or Abbott–Firestone parameters, but to provide complementary comparative information regarding roughness evolution, peak-height modification and material ratio preservation in worn FFF Kevlar-reinforced structures.

## 2. Materials and Methods

The present study builds upon the experimental framework previously reported in our earlier published work on FFF polymer structures and their tribological performance [9]. In that previous study, the analysis mainly focused on macroscopic tribological indicators, including friction behavior and overall wear performance. In contrast, the present investigation uses comparable specimen fabrication parameters and tribological testing conditions in order to ensure methodological consistency but extends the analysis toward quantitative profilometric characterization of wear-track geometry, volumetric material loss and surface integrity degradation.

### 2.1. Material and Specimen Manufacturing

The primary material investigated in this study was an aramid fiber-reinforced Acrylonitrile Styrene Acrylate (ASA) filament, commercially designated as ApolloX Kevlar (FormFutura^®^ 3D Printing Materials, Gelderland, The Netherlands) [27]. This structural composite was selected for its high impact and damage resistance, shatterproof properties and abrasive characteristics.

Specimens were manufactured using a Creality K1C FFF printer (Creality Co., Ltd., Shenzhen, China) and prior to extrusion, the hygroscopic filament was processed in a Creality Space Pi Filament Dryer (Creality Co., Ltd., Shenzhen, China) to prevent moisture-induced defects such as porosity or delamination.

The FFF process parameters used for manufacturing the Kevlar-reinforced ASA specimens were selected based on the material supplier recommendations and the previously reported experimental framework [9,27]. No additional process-parameter optimization was performed in the present study, since the objective was to maintain manufacturing consistency and enable direct comparison with the previously investigated tribological configurations. The extrusion temperature was set to 255 °C and the heated bed temperature to 90 °C. The printing speed was set to 25 mm/s, with a corresponding travel speed of 300 mm/s. The slicing parameters included a standard layer height of 0.20 mm, utilizing 2 perimeter walls, alongside 4 solid top and bottom layers. An Archimedean pattern was assigned to both the top and bottom solid surfaces [9]. For the partially filled specimens, the internal structure was defined by a gyroid sparse infill pattern manufactured at five infill density configurations: 10%, 30%, 50%, 70% and 90%. The 100% infill configuration did not preserve the gyroid sparse topology. Instead, it was manufactured as a fully dense reference specimen generated exclusively using the Archimedean pattern [9]. Therefore, the 100% configuration was considered as a solid reference condition rather than as a direct continuation of the gyroid infill series.

**Figure 1:**
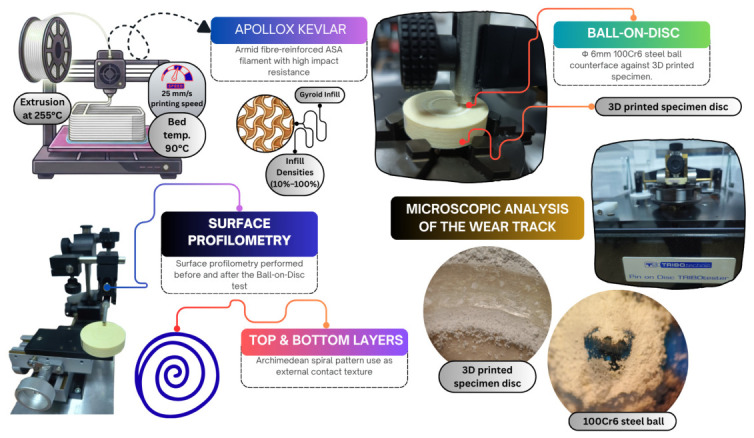
Experimental methodology for the tribological investigation of FFF ApolloX Kevlar specimens.

The 3D models were designed as cylindrical discs featuring a uniform diameter of 40 mm and a total height of 10 mm [9]. Before undergoing experimental evaluation, the surface of each FFF specimen was cleared of microparticles using compressed air and 99% isopropyl alcohol (IPA). The production and experimental workflow used for the FFF ApolloX Kevlar specimens is illustrated in Figure 1.

### 2.2. Tribological Testing

Tribological characterizations were executed using a TRIBOtester system configured as a Ball-on-Disc tribometer (Tribotechnic US LLC, San Francisco, CA, USA), strictly adhering to the ASTM G99-17 standard [28,29]. In this setup, the FFF polymer cylinder operated as the rotating disc, while the counterface consisted of a stationary 100Cr6 steel ball conforming to the DIN 17230 standard [30]. The spherical counterbody had a nominal diameter of 6 mm, a Young’s modulus of 205 GPa, and a Poisson’s ratio of 0.33. A new 100Cr6 steel ball was used for each individual Ball-on-Disc test in order to avoid the influence of counterbody wear, transferred polymer debris or pre-existing surface damage on subsequent tribological measurements.

A normal load of 5 N was applied vertically onto the rotating specimen, generating a computed Hertz pressure of 231 MPa. Testing occurred under dry sliding conditions without external lubrication [9]. The environment was rigidly controlled at an ambient temperature of 24 ± 1 °C and approximately 50% relative humidity in an air atmosphere. The sliding speed was set to 150 mm/s over a wear track radius of 9 mm, accumulating a total sliding distance of 300 m [9].

### 2.3. Surface Profilometry

Pre-test and post-test surface profilometry was carried out on the induced wear tracks using a stylus profilometer (Tribotechnic US LLC, San Francisco, CA, USA) equipped with a stylus DIAVITE 7891 (DIAVITE AG, Bülach, Switzerland) and a skid tracing system. The stylus was fitted with a standard diamond tip with a radius of 5 µm and a tip angle of 90°. The static measuring force was lower than 0.75 mN, while the static tracking force using the skid was lower than 0.15 N. The tracing speed was set to 0.5 mm/s. The instrument provided measurement ranges of 0–20.00 µm for *R_a_* and *R_q_* and 0–350.0 µm for the other measured profile parameters, with a readout resolution of 0.01 µm for all measured values. Three independent linear scans, each spanning a length of 4 mm, were acquired from each tested sample. The amplitude parameters were derived following the ISO 4287 standard [31], using a Gaussian profile filter with a cut-off wavelength of 0.8 mm. This filtering condition was selected to obtain the roughness profile by separating the shorter-wavelength roughness components from longer-wavelength waviness components, while maintaining consistent evaluation conditions for all unworn and worn profiles. Since each scan length was 4 mm, the selected cut-off provided five sampling lengths per profile, enabling direct comparison between the investigated specimens and infill configurations.

### 2.4. Wear Analysis

The net removed area (*A_net_*) was determined by subtracting the area outside the profile from the area of the wear hole. The overall wear volume (*V*) was calculated leveraging the geometrical relationship:

(1) $$V=A_{net}\cdot 2\pi R$$

where *R* defines the 9 mm track radius.

The specific wear rate (*k*) was computed using the equation:

(2) $$k=\frac{V}{\left(F\cdot L\right)}$$

with *F* corresponding to the normal load (5 N) and *L* representing the sliding distance (300 m).

A Wear Efficiency Index (*WEI*) was additionally evaluated, calculated as the inverse of the specific wear rate:

(3) $$WEI=\frac{1}{k}$$

To further compare the morphological evolution of the wear tracks as a function of internal infill architecture, the conventional profilometric parameters measured before and after tribological testing were expressed as relative changes. This approach was used because the absolute values of *R_a_*, *R_p_* and *R_mr_* describe the surface state at a given measurement stage, whereas the present study focuses on the relative transformation of the same surface from the unworn condition to the worn wear-track condition. Therefore, the following quantities should be interpreted as derived relative profilometric descriptors rather than as replacements for standardized roughness or Abbott–Firestone parameters. Their role is to provide a normalized comparative representation of pre-wear to post-wear surface evolution within the present experimental dataset.

The percentage variation in a profile parameter relative to the reference unworn state was calculated using:

(4) $$\Delta P=\frac{\bar{P_{w}}-\bar{P_{0}}}{\bar{P_{0}}}\cdot 100$$

where Δ*P* (%) represents the percentage variation in the surface parameter, $\bar{P_{w}}$ is the average value of the parameter measured on the wear track after the Ball-on-Disc test and $\bar{P_{0}}$ is the average initial value of the same parameter measured on the unworn surface before testing.

Based on this comparative approach, three derived relative descriptors were evaluated:

(5) $$STAI\left(\%\right)=\frac{\bar{R_{a_{w}}}-\bar{R_{a_{0}}}}{\bar{R_{a_{0}}}}\cdot 100$$

where *STAI* (%) represents the Surface Texture Alteration Index, $\bar{R_{a_{w}}}$ is the average value of the arithmetic mean roughness measured on the wear track after the Ball-on-Disc test and $\bar{R_{a_{0}}}$ is the average value of the arithmetic mean roughness measured on the initial unworn surface prior to testing.

(6) $$PDI(\%)=\frac{\bar{{R_{p}}_{w}}-\bar{{R_{p}}_{0}}}{\bar{{R_{p}}_{0}}}\cdot 100$$

where *PDI* (%) represents the Peak Deformation Index, $\bar{{R_{p}}_{w}}$ is the mean value of the maximum peak height measured within the wear track after testing and $\bar{{R_{p}}_{0}}$ is the mean value of the maximum peak height on the untreated surface before testing.

(7) $$MRPI=1-\frac{\left|\bar{R_{{mr}_{w}}}-\bar{R_{{mr}_{0}}}\right|}{\bar{R_{{mr}_{0}}}}$$

where *MRPI* is the Material Ratio Preservation Index, $\bar{R_{{mr}_{w}}}$ is the mean value of the material ratio parameter measured within the wear track after testing and $\bar{R_{{mr}_{0}}}$ is the mean value of the material ratio parameter before tribological testing. MRPI was introduced as a comparative descriptor of the relative preservation of the material ratio after wear. Higher MRPI values indicate a smaller relative deviation from the initial *R_mr_* value, whereas lower values indicate a stronger alteration of the material ratio within the wear track.

### 2.5. Optical Microscopy Analysis

Topographical transformations and wear mechanisms were visually inspected using a digital stereo optical microscope (NOVEX RZT-SF, Euromex, Duiven, The Netherlands) equipped with built-in directed lighting. The microscope was outfitted with a CMEX DC.1300 × USB camera and connected to ImageFocus v2.5 software for direct image acquisition.

### 2.6. Statistical Analysis

Three separate specimens were manufactured and analyzed for each designated infill density configuration to provide an initial estimate of experimental variability through mean and standard deviation values. Quantitative data extracted from the wear testing and profilometry were synthesized using the arithmetic mean and standard deviation formulas. To assess the influence of the internal gyroid infill percentage on the dependent variable of specific wear rate, a one-way analysis of variance (ANOVA) was executed, alongside computations of the effect size (*η*^2^). The associations between parameters, such as maximum wear depth and removed material volume, were evaluated using the Pearson correlation coefficient (*r*), while linear regression was applied to determine the coefficient of determination (*R*^2^).

## 3. Results

This section presents the experimental results regarding the tribological performance and surface morphological evolution of the FFF Kevlar-reinforced composite specimens as a function of the internal gyroid infill density.

### 3.1. Wear Volume and Specific Wear Rate

The tribological performance of the FFF Kevlar-reinforced composite specimens was quantitatively evaluated by calculating the removed wear volume (*V*), the specific wear rate (*k*) and the wear efficiency index (*WEI*). The calculated parameters across all investigated gyroid infill densities are summarized in Table 1.

The experimental data indicate that the variations in wear volume and specific wear rate as a function of infill density follow a non-monotonic trend. Within the tested conditions, the 30% infill density configuration showed the most favorable average wear response, recording the lowest average wear volume (7.45 mm^3^) and the lowest specific wear rate (4.96 × 10^−3^ mm^3^/N·m), which corresponded to the highest Wear Efficiency Index (201.34 N·m/mm^3^). Therefore, the 30% infill configuration is discussed as the configuration with the most favorable average wear response under the present experimental conditions. On the other hand, the sample with infill density of 90% exhibited the lowest wear resistance, yielding the highest average wear volume (10.3 mm^3^) and the highest specific wear rate (6.86 × 10^−3^ mm^3^/N·m), resulting in the lowest Wear Efficiency Index (145.57 N·m/mm^3^).

The observed non-monotonic behavior can be interpreted as the result of a balance between internal support stiffness, local compliance of the printed mesostructure and debris retention within the contact region. At low infill density, the reduced internal support may promote local deformation of the upper printed layers under the 100Cr6 steel counterbody, facilitating deeper penetration and material removal. At intermediate infill density, the gyroid architecture may provide a more favorable compromise between load support and local deformation accommodation, allowing the contact stresses to be distributed without excessive collapse of the surface layers. At higher infill densities, the increased structural stiffness may reduce the ability of the mesostructure to accommodate contact-induced deformation, promoting more severe ploughing, debris compaction and third-body abrasion within the wear track. This interpretation is consistent with the high wear volume observed for the 90% infill configuration and with the optical evidence of extensive debris accumulation and counterbody scratching observed after testing.

**Figure 2:**
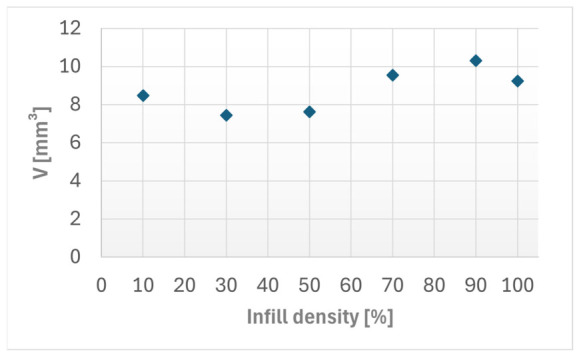
Wear volume (*V*) as a function of infill density for the FFF Kevlar-reinforced ASA specimens. The linear regression line and coefficient of determination (*R*^2^) are indicated.

Figure 2 below illustrates the relation between the wear volume and the infill density among the tested specimens. The results reveal changes in the infill densities, where the lowest wear volume occurred at 30% infill density, while the highest one was observed at 90% infill density.

**Figure 3:**
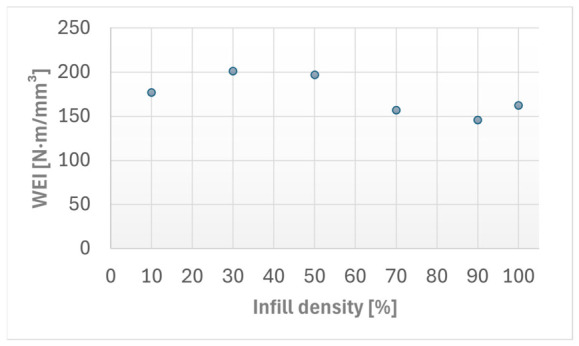
Wear Efficiency Index (*WEI*) as a function of infill density for the FFF Kevlar-reinforced composite specimens. The linear regression fit and the corresponding coefficient of determination (*R*^2^) are indicated.

Figure 3 illustrates the dependence of the Wear Efficiency Index (*WEI*) on the infill density of the specimens. The results have shown that the variations across the configurations investigated, with higher *WEI* values, correspond to improved wear resistance.

Across the tested spectrum, all measured specific wear rates were on the order of 10^−3^ mm^3^/N·m, indicating a relatively high wear rate for the investigated polymer composite. The absolute range of variation for the specific wear rate $\left(k_{max}-k_{min}\right)$ across the different internal architectures was approximately 19.02 × 10^−4^ mm^3^/N·m. Analysis of the ratio between these extreme values shows that the 90% infill configuration exhibited approximately 38% higher wear than the 30% infill configuration. Overall, the average wear response improved from 10% infill to the 30% infill and subsequently deteriorated as infill density increased through 50%, 70% and 90%, before exhibiting a slight final recovery for the fully solid 100% infill condition (6.16 × 10^−3^ mm^3^/N·m, *WEI* 162.28 N·m/mm^3^). However, this configuration should be interpreted as a fully dense reference condition rather than as a direct continuation of the partially filled gyroid series.

**Figure 4:**
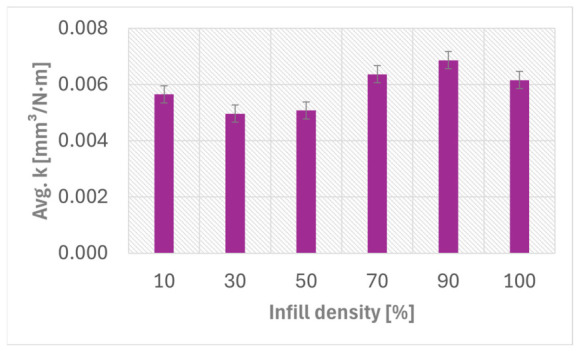
The Average specific wear rate (*k*) versus FFF Kevlar-reinforced ASA composite specimens infill densities.

The variation in the average specific wear rate ($\bar{k}$) with infill density is presented in Figure 4. The values were calculated from the profilometric determined wear volumes obtained after the Ball-on-Disc tests.

### 3.2. Wear Track Profilometry

To quantitatively evaluate the surface damage induced during the dry sliding Ball-on-Disc tests, post-test profilometric measurements were conducted across the generated wear tracks. For each Kevlar-reinforced FFF specimen, three independent linear stylus scans, each spanning a length of 4 mm, were acquired transversally across the sliding path to assess the cross-sectional wear profile and ensure data reliability. All the average values of the maximum profile height ($R_{z}$), maximum valley depth ($R_{v}$) and reduced roughness depth ($R_{dc}$) were reported for each investigated infill density before and after the Ball-on-Disc in Table 2.

The percentage variation (Δ*P*) of the main profilometric surface parameters after the tribological tests is presented in Table 3.

**Figure 5:**
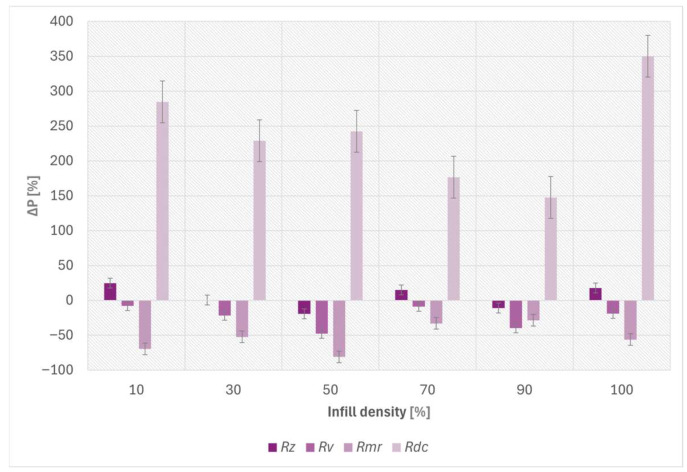
Percentage variation (Δ*P*) of the main profilometric surface parameters (*R_z_*, *R_v_*, *R_mr_*, *R_dc_*) calculated from the values measured before and after the Ball-on-Disc tests for the investigated infill densities.

Figure 5 illustrates the percentage variation (Δ*P*) of the profilometric parameters *R_z_*, *R_v_*, *R_mr_*, *R_dc_* for the investigated infill densities.

**Figure 6:**
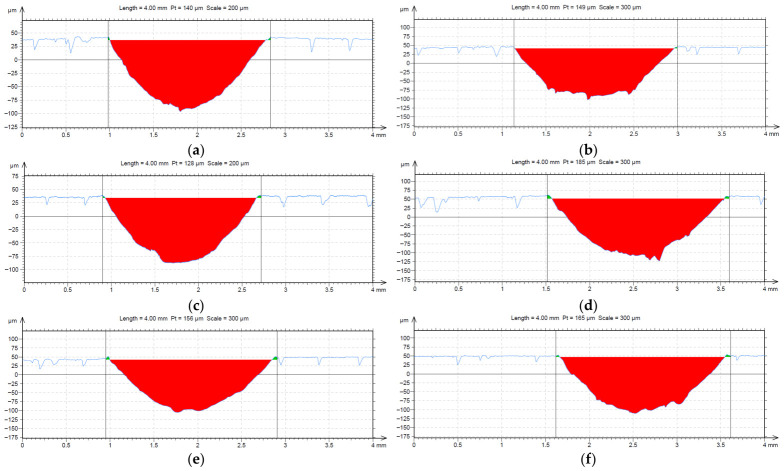
2D wear-track profiles obtained after the Ball-on-Disc tests on the FFF Kevlar-reinforced ASA composite specimens investigated in this study (the blue line represents the measured surface profile, while the green color indicates the boundaries of the selected measurement area): (**a**) 10%; (**b**) 30%; (**c**) 50%; (**d**) 70%; (**e**) 90%; (**f**) 100%.

Figure 6 presents representative cross-sectional profilometric profiles of the wear tracks produced during the tribological tests for the different infill densities. The red shaded regions represent the removed material area within the wear track.

The profilometric results revealed distinct variations in the maximum wear-track depth as a function of the internal gyroid infill density. The 30% infill sample showed the lowest average wear depth value of 120.37 µm, followed closely by the 50% infill sample with 121.33 µm average wear depth. The deepest wear tracks were found in the 70% and 90% infill samples, with average depths of 154.33 µm and 153.67 µm, respectively. These differences indicate that wear-track geometry was not governed only by the nominal contact conditions, but also by the ability of the internal architecture to support the upper printed layers during sliding contact.

**Figure 7:**
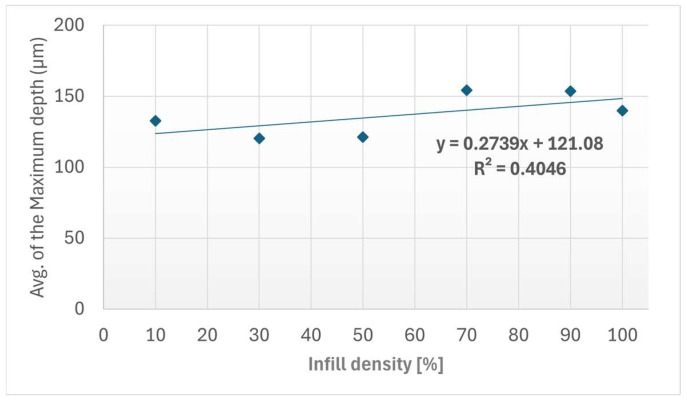
Maximum wear-track depth as a function of infill density obtained from stylus profilometry measurements for the FFF Kevlar-reinforced composite specimens. The linear regression fit and the coefficient of determination (*R*^2^) are indicated.

The variation in the maximum wear-track depth with infill density is presented in Figure 7. The values were extracted from the cross-sectional profilometric measurements of the wear tracks obtained after the Ball-on-Disc tests. From a mechanistic perspective, the shallower wear tracks observed for the 30% and 50% infill configurations may be associated with a more favorable balance between local compliance and structural support beneath the contact zone. In these configurations, the gyroid mesostructure may allow partial accommodation of the contact-induced deformation while still limiting excessive collapse of the top layers. Conversely, the larger wear-track depths observed for the 70% and 90% infill configurations suggest a less favorable stress accommodation condition, where the increased internal stiffness may promote more concentrated ploughing and material removal at the surface. In addition, the heterogeneous Kevlar-reinforced ASA microstructure may contribute to local variations in wear-track geometry through fiber exposure, matrix removal and debris entrapment within the sliding path.

The acquired cross-sectional profilograms display characteristic U-shaped valleys that mirror the geometry of the 6 mm spherical steel counterbody. An examination of the representative wear profiles highlights the geometrical differences across infill configurations. The profiles extracted from the 30% infill specimens showed a shallower and narrower wear scar, corresponding to the lowest average net removed cross-sectional area (*A_net_*) of 0.132 mm^2^. In contrast, the profiles corresponding to the 90% infill density presented wider and deeper wear tracks, reflecting more pronounced geometric deformation and the highest average removed cross-sectional area of 0.183 mm^2^.

These cross-sectional profilometric parameters are mathematically linked to the overall material loss presented in Section 3.1. The calculated wear volume (*V*) was derived directly from the profilometrically measured net removed area (*A_net_*) utilizing the geometric Equation (1), where *R* is the 9 mm track radius. A statistical evaluation of the experimental data confirmed a very strong positive correlation (Pearson coefficient *r* = 0.967) between the maximum wear-track depth and the computed removed material volume. Furthermore, simple linear regression between these two parameters yielded a high coefficient of determination (*R*^2^ = 0.935). This confirms that the calculated volumetric wear is strongly associated with the physical depth and cross-sectional shape of the localized wear track induced by the sliding counterface.

### 3.3. Profilometric Wear Indices

#### 3.3.1. Surface Texture Alteration Index

The Surface Texture Alteration Index (STAI) was used as a derived relative descriptor calculated from the standardized arithmetic mean roughness parameter (*R_a_*) of the surface. It does not replace *R_a_* and should not be interpreted as an independent roughness parameter. Instead, STAI expresses the relative pre-wear to post-wear change in *R_a_* in percentage form, allowing the roughness alteration of specimens with different initial surface states to be compared within the same experimental dataset. The STAI values calculated from the arithmetic mean roughness measured before and after the Ball-on-Disc tests are summarized in Table 4.

It should be noted that STAI is not intended to rank the overall wear severity or to directly predict the wear volume or specific wear rate. Instead, it was retained as a complementary descriptor because it isolates the relative change in the arithmetic mean roughness *R_a_* from the broader volumetric wear response. Since STAI is normalized by the initial *R_a_* value, it is sensitive to the initial roughness state and to changes in the average height distribution of the profile, but it does not directly account for wear-track depth, cross-sectional removed area or debris accumulation. For example, the 90% infill configuration exhibited a relatively low STAI value because its initial *R_a_* was the highest among the investigated specimens, and the post-wear increase in *R_a_* was proportionally limited. However, this does not contradict its high wear volume and specific wear rate, which were primarily reflected by the deeper and wider wear-track geometry and the higher removed material volume. Therefore, STAI should be interpreted as a complementary descriptor of relative roughness alteration, rather than as a standalone indicator of tribological wear resistance.

#### 3.3.2. Peak Deformation Index

The Peak Deformation Index (PDI) was used as a derived relative descriptor calculated from the standardized maximum profile peak height parameter (*R_p_*). PDI does not replace *R_p_* and should not be interpreted as an independent surface parameter or as a direct indicator of global wear resistance. Instead, it expresses the relative pre-wear to post-wear change in *R_p_* in percentage form, allowing the modification of peak-height features to be compared between specimens with different initial surface states. The PDI values derived from the average maximum peak height measured before and after the Ball-on-Disc tests are presented in Table 5.

The highest PDI values were obtained for the 100% and 10% infill configurations, with values of 205.42% and 190.70%, respectively. The 50% infill configuration showed an intermediate PDI value of 146.20%, whereas the 90% and 30% configurations showed similar values of 121.47% and 120.32%, respectively. These results indicate that PDI does not follow the same trend as wear volume or specific wear rate. This is expected because PDI is derived only from the relative change in *R_p_* and therefore reflects the evolution of localized peak-height features rather than the total amount of material removed. High PDI values may indicate the formation or persistence of localized protrusions within the worn profile, which can result from asperity deformation, exposed fibers, compacted debris or local material redistribution. From a mechanistic perspective, the post-wear increase in *R_p_* may result from the formation of localized protrusions within or near the wear track, caused by plastic deformation of asperities, partial ridge collapse, exposure or pull-out of Kevlar fibers, compacted debris and local material redistribution during repeated sliding contact. Therefore, PDI should be interpreted as a complementary descriptor of peak-height modification, rather than as a standalone indicator of tribological wear performance.

#### 3.3.3. Material Ratio Preservation Index

The Material Ratio Preservation Index (MRPI) was used as a derived relative descriptor calculated from the Abbott–Firestone material ratio parameter $R_{mr}$. MRPI does not replace $R_{mr}$ and should not be interpreted as an absolute measure of surface integrity. Instead, it expresses the relative preservation of the material ratio after wear, allowing the pre-wear to post-wear modification of load-bearing profile characteristics to be compared within the present experimental dataset. The MRPI values derived from the average material ratio measured on the unworn surface and within the wear track after the Ball-on-Disc tests are presented in Table 6.

The calculated MRPI values varied across the investigated infill densities, with higher values obtained for the 90% and 70% configurations and lower values for the 10% and 50% configurations. However, these values should be considered in relation to the relatively large baseline variability of the material ratio ($R_{{mr}_{0}}$) observed for several infill configurations. This variability reflects the heterogeneous initial surface state of the FFF specimens, including the influence of printed ridges, valleys, local voids and fiber-related surface irregularities. Since MRPI is normalized by $R_{{mr}_{0}}$, variability in the baseline material ratio can directly influence the calculated preservation index. Therefore, MRPI is used here as a complementary comparative descriptor of relative material ratio preservation, rather than as a standalone or absolute ranking of surface integrity.

#### 3.3.4. Correlation Analysis of Wear Parameters

A statistical correlation analysis was conducted to quantify the relationships between the principal tribological parameters, infill density and the initial surface topography of the tested specimens. The associations were evaluated using the Pearson correlation coefficient (*r*) and simple linear regression to determine the coefficient of determination (*R*^2^).

**Figure 8:**
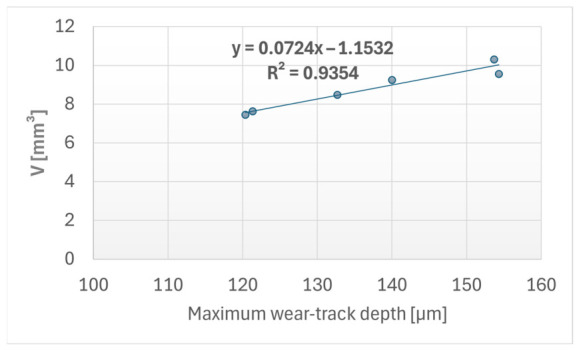
The correlation between maximum wear-track depth and wear volume.

The relationship between the geometric dimensions of the wear track and the overall material loss was first evaluated. A very strong positive correlation was observed between the maximum wear-track depth and the computed wear volume, yielding a Pearson coefficient of *r* = 0.967. The linear regression analysis for these parameters produced a high coefficient of determination (*R*^2^ = 0.935), indicating a strong statistical association between wear-track depth and calculated wear volume. However, this correlation should not be interpreted as direct evidence of a cause-and-effect relationship. Rather, it indicates that deeper wear tracks were consistently associated with higher calculated material loss within the present experimental dataset. The relationship between the maximum wear-track depth and the calculated wear volume is presented in Figure 8.

**Figure 9:**
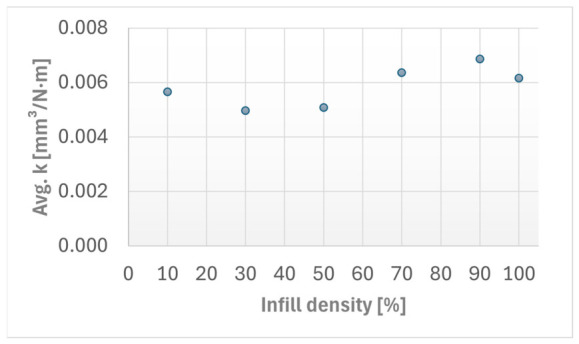
The correlation between infill density and specific wear rate.

The effect of the internal structural architecture on the resulting wear behavior was assessed by correlating the gyroid infill density percentage with the specific wear rate (*k*). The analysis revealed a strong positive correlation between these variables (*r* = 0.705). The linear regression model produced an *R*^2^ value of 0.497, indicating that approximately 49.7% of the variability in the specific wear rate within the present dataset was associated with the variation in infill density. The relationship between the average specific wear rate (*k*) and the infill density is illustrated in Figure 9.

Finally, the influence of the initial surface topography on the subsequent specific wear rate (*k*) was statistically evaluated using three pre-wear profilometric parameters: initial arithmetic mean roughness (${R_{a}}_{0}$), initial material ratio (${R_{mr}}_{0}$), and initial profile section height difference (${R_{dc}}_{0}$).

**Figure 10:**
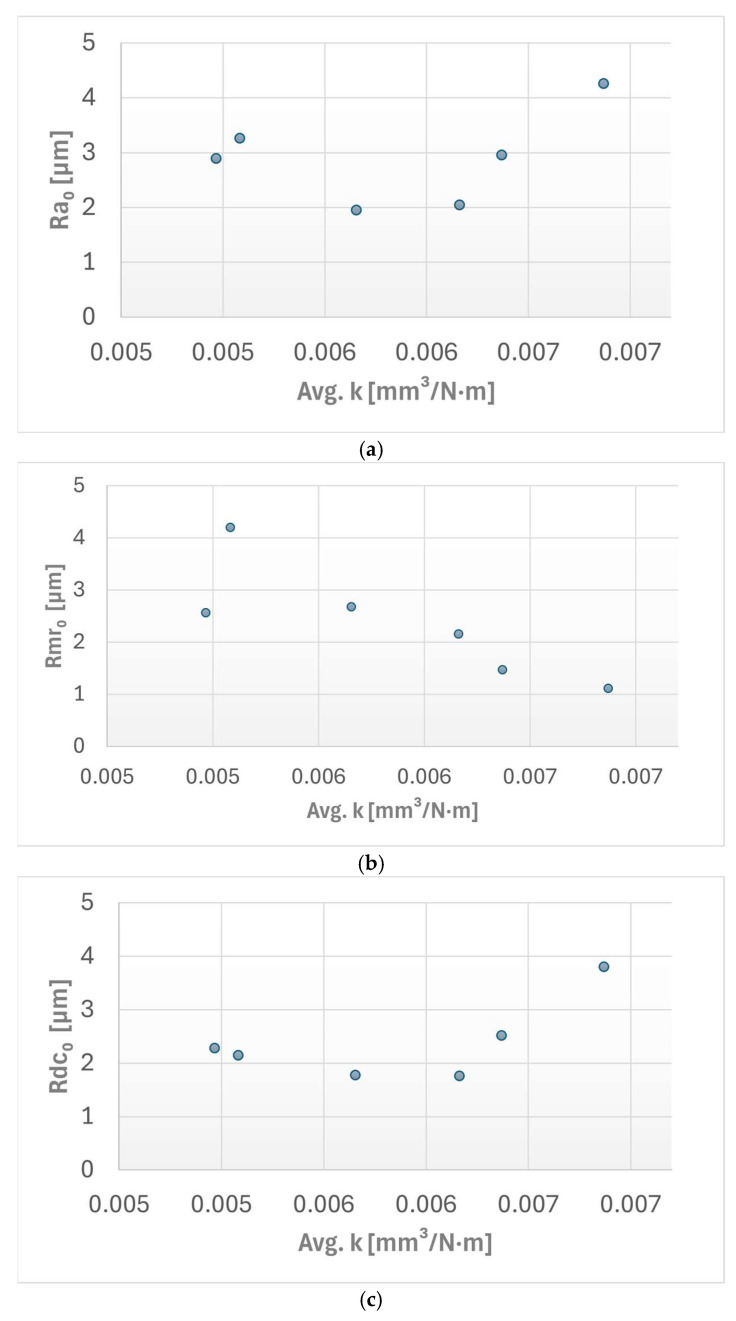
The correlation between the average specific wear rate and the selected surface profilometric parameters: (**a**) the arithmetic mean roughness ${R_{a}}_{0}$; (**b**) the material ratio ${R_{mr}}_{0}$; (**c**) the reduced roughness depth ${R_{dc}}_{0}$.

The correlations between the average specific wear rate (*k*) and selected profilometric surface parameters are illustrated in Figure 10.

For the arithmetic mean roughness (${R_{a}}_{0}$) vs. specific wear rate (*k*), a weak positive correlation was identified, with a recording Pearson coefficient of *r* = 0.332 and a low coefficient of determination value of *R*^2^ = 0.1104.

For the material ratio (${R_{mr}}_{0}$) vs. specific wear rate (*k*): a very strong negative correlation was observed between the initial material ratio and the resulting wear rate. The statistical analysis resulted in a Pearson coefficient of *r* = −0.846 and a strong coefficient of determination of *R*^2^ = 0.7151.

In the profile section height difference (${R_{dc}}_{0}$) vs. specific wear rate (*k*) case this relationship showed a strong positive correlation, with a Pearson coefficient of *r* = 0.606 and a moderate coefficient of determination of *R*^2^ = 0.3672.

A one-way ANOVA was performed to quantitatively evaluate the influence of the internal gyroid infill percentage on the resulting specific wear rate (*k*). The statistical comparison included the six investigated infill density configurations. The results did not reveal statistically significant differences at *α* = 0.05, F(5,12) = 1.679 and *p* = 0.214. Therefore, a statistically confirmed effect of infill density on the specific wear rate could not be established within the present dataset. However, the calculated effect size (*η*^2^ = 0.412) suggests a potentially relevant practical trend, indicating that internal architecture may contribute to the observed variability in wear response. Accordingly, the effect-size result is interpreted as an indicative practical trend rather than as statistically confirmed evidence of a reliable group difference.

### 3.4. Optical Microscopy Analysis of Wear-Track Morphology

After the Ball-on-Disc tribological testing, optical microscopy of the FFF Kevlar-reinforced specimens revealed pronounced surface degradation characterized by the formation of continuous, deep wear channels. Across all investigated gyroid infill densities (10% to 100%), the sliding action of the 100Cr6 steel counterbody substantially altered the original Archimedean top-layer pattern, cutting distinct grooves into the upper layers of the printed structures.

A defining morphological feature consistently observed across all Kevlar specimens was the extensive generation and accumulation of wear debris. Microscopic evaluations showed extensive amounts of white polymeric dust, comprising both fine particulates and larger agglomerates, packed tightly within the wear tracks and piled heavily along the track margins.

The marked severity of the sliding contact was further evidenced by the condition of the 100Cr6 steel counterbodies. Optical inspection of the spherical pins revealed significant amounts of the white Kevlar composite material adhered directly to the contact area. Following the removal of these adhered residues, clear directional wear scars and distinct scratching marks were visible on the steel balls themselves across all tested infill configurations.

**Figure 11:**
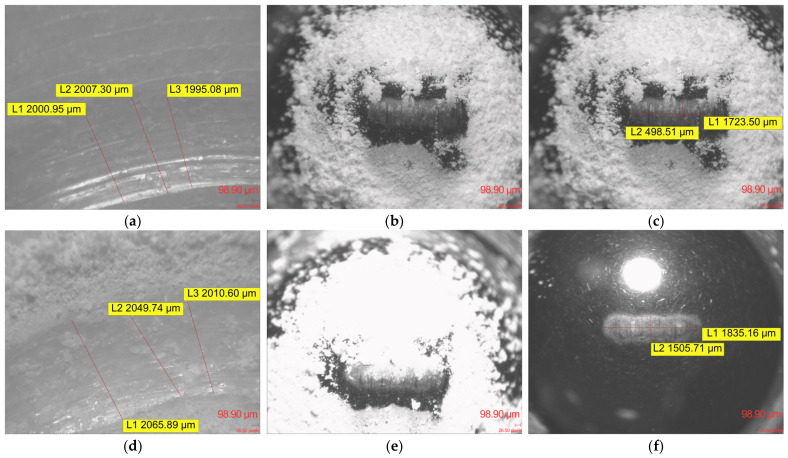
Optical microscopy images of wear-track morphology and counterbody surface after Ball-on-Disc testing of ApolloX Kevlar specimens: (**a**) wear track on specimen with 30% gyroid infill; (**b**) 100Cr6 steel ball covered with ApolloX Kevlar debris after testing against the 30% infill specimen; (**c**) wear traces on the steel ball after removal of adhered debris; (**d**) wear track on specimen with 50% gyroid infill; (**e**) steel ball covered with transferred polymer debris after testing against the 50% infill specimen; (**f**) wear traces on the steel ball after removal of adhered debris.

Despite variations in the internal gyroid infill density, the macroscopic characteristics of the surface damage, specifically the deep ploughed profiles, extensive white debris formation and visible abrasive scarring of the steel counterface, remained uniform throughout the Kevlar specimen series. Figure 11 presents optical microscopy images of the wear-track morphology and the corresponding counterbody surfaces after the Ball-on-Disc tests for the investigated ApolloX Kevlar specimens.

## 4. Discussion

The tribological and structural evaluation of the FFF Kevlar-reinforced composites revealed a complex, non-monotonic relationship between internal gyroid infill density and the resulting macroscopic wear behavior. The experimental data showed that, within the tested conditions, the 30% infill density configuration exhibited the most favorable average wear response, yielding the lowest removed wear volume and specific wear rate, which inherently corresponded to the highest Wear Efficiency Index (*WEI*). Conversely, denser structures such as the 90% infill configuration exhibited the highest material loss. Within the limits of the present experimental dataset, the 30% infill configuration therefore represented the tested condition with the lowest average wear volume and specific wear rate. This non-linear trend indicates that the tribological response of the composites is not governed solely by the bulk density of the material. Instead, the macroscopic wear response may be influenced by the combined effect of the internal load-bearing framework, the local compliance of the mesostructure and the ability of the printed architecture to accommodate contact-induced deformation during sliding. Across all configurations, the specific wear rate remained in the order of 10^−3^ mm^3^/N·m, indicating a relatively high wear level under the present dry sliding conditions and providing the basis for comparison with previously reported FFF polymer systems. While direct numerical comparison is limited by differences in testing conditions, such as normal load, sliding speed, test geometry and FFF parameters, the specific wear rates obtained in the present study are of the same order of magnitude as values reported by Abed et al. for additively manufactured PLA [32]. These values indicate substantial material removal under the present dry sliding conditions and are consistent with previously reported observations that FFF polymer wear is strongly influenced by internal architecture, reinforcement and processing parameters. For instance, Ergene et al. showed that volume loss in PLA and carbon fiber-reinforced PLA can vary significantly with infill density [14], supporting the relevance of structural parameters for wear resistance. Similar behavior was also reported for comparable FFF polymer systems, where reinforced filaments such as ApolloX Kevlar and PLA-CF exhibited pronounced abrasive wear, deep wear profiles and debris formation under sliding conditions [9]. This interpretation is further supported by the fact that rigid fillers or detached reinforcement fragments may act as abrasive third bodies when liberated from the polymer matrix during sliding contact [1]. Therefore, although the present results reflect specific localized contact conditions, the measured wear level and the observed wear mechanisms are broadly consistent with expected behavior for reinforced thermoplastic FFF components. The volumetric material loss of the specimens was found to be strongly associated with the cross-sectional deformation of the wear track. A very strong positive correlation (*r* = 0.967) and a high coefficient of determination (*R*^2^ = 0.935) were identified between the maximum wear-track depth and the overall calculated wear volume. This relationship should not be interpreted as direct evidence of a cause-and-effect mechanism, but rather as a strong statistical association within the present experimental dataset. The results indicate that deeper and wider wear tracks were consistently associated with higher calculated material loss. Accordingly, the wear-track geometry provides an important geometrical descriptor of the volumetric degradation, while the underlying wear mechanisms should be interpreted together with the profilometric and optical microscopy observations.

The observed material loss appears to be associated with combined abrasive and adhesive wear processes involving the polymer matrix, Kevlar reinforcement and third-body debris. While Kevlar reinforcements are typically incorporated to enhance impact resistance and structural stiffness, the optical microscopy observations revealed white debris accumulation, deep ploughed profiles and scratching marks on the 100Cr6 steel counterbody. These features suggest the occurrence of abrasive ploughing, material transfer and debris-mediated sliding rather than proving a single dominant wear mechanism. During sliding, the polymer matrix may be progressively removed or redistributed, exposing fiber-rich regions and generating debris within the contact interface. Detached fibers, fragmented matrix material and compacted debris may then contribute to third-body abrasion and localized scratching of the steel counterbody. Therefore, the proposed wear interpretation should be regarded as a mechanism consistent with the observed profilometric and optical evidence, rather than as a directly proven microscopic sequence.

To compare the topographical changes induced by sliding, this study used three derived relative profilometric descriptors: STAI, PDI and MRPI. These descriptors are not proposed as replacements for standardized roughness or Abbott–Firestone parameters, but as complementary comparative quantities calculated from conventional profilometric measurements. Their purpose is to describe specific aspects of the pre-wear to post-wear surface transformation, namely relative roughness alteration, peak-height modification and material ratio preservation. The use of these descriptors supports a multidimensional interpretation of wear-track degradation, because the evolution of roughness, peak morphology and material ratio does not necessarily follow the same trend as wear volume or specific wear rate. For example, PDI should be interpreted as a descriptor of localized peak-height modification rather than as a direct measure of global wear severity. The post-wear increase in *R_p_* may be associated with localized protrusions, compacted debris, exposed fibers or local material redistribution within the wear track, but these mechanisms require cautious interpretation and should be considered together with the optical observations.

The relationship between the initial FFF surface topography and the subsequent wear response was evaluated through correlation analysis. Conventional arithmetic mean roughness ($R_{a_{0}}$) exhibited only a weak positive correlation (*r* = 0.332) with the specific wear rate, suggesting that baseline *R_a_* alone was not sufficient to explain the wear response of these structured FFF surfaces. Conversely, the initial material ratio ($R_{{mr}_{0}}$) showed a very strong negative correlation (*r* = −0.846) with the specific wear rate. This statistical relationship suggests that specimens with higher initial material ratio values tended to exhibit lower specific wear rates within the present dataset. However, this correlation should not be interpreted as direct causal proof that $R_{{mr}_{0}}$ alone controls the wear response. Instead, it indicates that the initial load-bearing profile characteristics may be relevant for understanding how the surface accommodates the imposed Hertzian contact. In addition, the positive correlation observed for the reduced roughness depth ($R_{{dc}_{0}}$) suggests that deeper initial profile features may be associated with increased susceptibility to localized deformation and material removal, although this interpretation remains based on statistical association.

The statistical analysis of the internal architecture also requires a clear distinction between observed experimental trends and statistically confirmed group differences. The one-way ANOVA evaluating the influence of infill density on specific wear rate did not reveal statistically significant differences at the conventional threshold (*p* = 0.214). Therefore, a statistically confirmed effect of infill density on specific wear rate could not be established within the present dataset. The effect size (*η*^2^ = 0.412) suggests a potentially relevant practical trend, indicating that internal architecture may contribute to the observed variability in wear response. Thus, the effect-size result supports the observed practical trend, but it does not confirm a reliable group difference in the absence of statistical significance. Accordingly, the differences between infill configurations are discussed in relation to the measured trends and supporting profilometric observations.

These findings are consistent with previous observations in AM tribology showing that reinforced polymer systems may exhibit complex wear behavior due to the interaction between matrix deformation, reinforcement exposure, debris formation and surface texture evolution. In the present study, the combination of wear volume, specific wear rate, wear-track geometry, optical microscopy and relative profilometric descriptors indicates that the degradation of FFF Kevlar-reinforced ASA surfaces is multidimensional. The high wear depths and the presence of transferred material and counterbody scratching support the interpretation that debris-mediated abrasion and localized ploughing contributed to the observed damage. However, the optical observations are qualitative, and therefore the proposed mechanisms should be regarded as interpretations consistent with the available evidence, rather than as direct microscopic confirmation of all individual wear processes.

The main contribution of this study is therefore not the replacement of standardized roughness descriptors, but the integration of conventional profilometric measurements into a comparative pre-wear/post-wear framework for evaluating wear-track degradation in FFF Kevlar-reinforced ASA structures. By combining wear volume, specific wear rate, wear-track depth, cross-sectional removed area, STAI, PDI and MRPI, the study provides a complementary metrological description of surface degradation across different internal architectures. This approach helps distinguish between volumetric material loss, roughness alteration, peak-height modification and material ratio preservation, which are not necessarily captured by a single parameter.

Ultimately, the findings have practical implications for the tribological design of FFF Kevlar-reinforced polymer structures. Within the present experimental conditions, the 30% infill configuration showed the most favorable average wear response, while the 90% infill configuration showed the highest material loss. These results suggest that intermediate infill architectures may provide a favorable balance between structural support and local deformation accommodation under dry sliding contact. Nevertheless, this conclusion should be understood as dataset-specific and should be further validated using larger sample sizes, additional loading conditions and complementary microscopic characterization. From a design perspective, improving the initial load-bearing surface profile and controlling the internal architecture may contribute to reducing localized wear-track degradation in aramid-reinforced FFF components.

## 5. Conclusions

This study evaluated the tribological performance and topographical degradation of FFF Kevlar-reinforced ASA composites using a comparative profilometric framework based on pre-wear and post-wear surface measurements. The macroscopic wear behavior exhibited a non-monotonic dependence on the internal gyroid infill density, with intermediate architectures, specifically 30% infill configuration showing the most favorable average wear response under the present experimental conditions. The 30% infill configuration recorded the lowest wear volume and specific wear rate, whereas the 90% infill configuration showed the highest material loss. These results suggest that the tribological response may be influenced by the combined effect of internal structural support, local compliance and contact-induced deformation accommodation. A very strong statistical correlation was established between the maximum wear-track depth and volumetric material loss, showing that deeper wear-track profiles were consistently associated with higher calculated material removal within the investigated dataset, without implying a direct cause-and-effect relationship. The derived relative profilometric descriptors, Surface Texture Alteration Index (STAI), Peak Deformation Index (PDI) and Material Ratio Preservation Index (MRPI), were used as complementary comparative parameters calculated from standardized roughness and Abbott–Firestone-based measurements. These descriptors do not replace conventional parameters, but provide additional comparative information regarding relative roughness alteration, peak-height modification and material ratio preservation after wear. Their interpretation should be considered together with wear volume, specific wear rate, wear-track geometry and optical microscopy observations. The correlation results suggest that the initial material ratio $R_{{mr}_{0}}$ may be more closely associated with the subsequent wear response than the initial arithmetic mean roughness $R_{a_{0}}$ within the present dataset. Although the differences in specific wear rate among infill configurations were not statistically significant, the observed wear trends suggest that internal architecture may still be relevant to the tribological response of the investigated specimens. Therefore, the influence of infill configuration should be interpreted as an observed experimental trend supported by profilometric evidence, not as a statistically confirmed group difference. Ultimately, these findings suggest that optimizing both the initial load-bearing surface topography and the internal gyroid architecture may contribute to reducing localized wear-track degradation and improving the functional durability of additively manufactured Kevlar-reinforced components in sliding applications. Further work involving larger sample sizes, additional loading conditions and complementary microscopic characterization would be useful to validate the observed trends and refine the interpretation of the wear mechanisms.

## Figures and Tables

**Table 1:** Wear volume, specific wear rate and derived wear parameters as a function of infill density.

Infill [%]	Avg. Area of the Hole [mm^2^]	Avg. Area Outside [mm^2^]	$\bar{A_{net}}$ ^1^ [mm^2^]	$\bar{V}$ ^2^ [mm^3^]	$\bar{k}$ ^3^ [mm^3^/N·m]	*WEI* ^4^ [N·m/mm^3^]
10	15.01 × 10^−2^	10.40 × 10^−5^	14.99 × 10^−2^	8.48	5.65 × 10^−3^	176.83
30	13.18 × 10^−2^	15.60 × 10^−5^	13.17 × 10^−2^	7.45	4.96 × 10^−3^	201.34
50	13.50 × 10^−2^	27.60 × 10^−5^	13.48 × 10^−2^	7.62	5.08 × 10^−3^	196.75
70	16.92 × 10^−2^	26.08 × 10^−5^	16.89 × 10^−2^	9.55	6.36 × 10^−3^	157.01
90	18.26 × 10^−2^	45.70 × 10^−5^	18.22 × 10^−2^	10.30	6.86 × 10^−3^	145.57
100	16.37 × 10^−2^	25.96 × 10^−5^	16.34 × 10^−2^	9.24	6.16 × 10^−3^	162.28

^1^ $\bar{A_{net}}$ represents the average net removed cross-sectional area of the wear track, calculated as the difference between the hole area and the area outside the wear profile. ^2^ $\bar{V}$ was obtained from profilometric measurements using Equation (1). ^3^ $\bar{k}$ was calculated using Equation (2). ^4^ WEI was calculated using Equation (3).

**Table 2:** Average surface roughness parameters measured before and after the Ball-on-Disc test.

Infill [%]	$\bar{{R_{z}}_{0}}$ ^1^ ± *SD* [µm]	$\bar{{R_{z}}_{w}}$ ^2^ ± *SD* [µm]	$\bar{{R_{v}}_{0}}$ ^3^ ± *SD* [µm]	$\bar{{R_{v}}_{w}}$ ^4^ ± *SD* [µm]	$\bar{{R_{dc}}_{0}}$ ^5^ ± *SD* [µm]	$\bar{{R_{dc}}_{w}}$ ^6^ ± *SD* [µm]
10	18.97 ± 1.99	23.67 ± 2.04	15.83 ± 2.19	14.60 ± 1.00	1.78 ± 0.53	6.85 ± 0.44
30	25.47 ± 2.56	25.63 ± 6.34	21.50 ± 1.93	16.83 ± 4.11	2.28 ± 0.36	7.50 ± 0.46
50	26.87 ± 4.83	21.73 ± 3.51	22.93 ± 3.95	11.99 ± 2.96	2.14 ± 0.63	7.35 ± 0.34
70	24.80 ± 6.76	28.57 ± 4.59	19.93 ± 5.17	18.17 ± 3.74	2.52 ± 0.76	6.98 ± 2.14
90	33.00 ± 2.55	29.40 ± 5.90	27.10 ± 2.52	16.33 ± 2.23	3.81 ± 0.69	9.43 ± 2.03
100	21.40 ± 5.39	25.23 ± 3.00	17.90 ± 4.80	14.53 ± 2.03	1.76 ± 0.14	7.92 ± 1.20

^1^ $\bar{{R_{z}}_{0}}$ represents the average maximum height of profile before tribological testing. ^2^ $\bar{{R_{z}}_{w}}$ is the average maximum height of profile after the Ball-on-Disc testing. ^3^ $\bar{{R_{v}}_{0}}$ represents the average maximum profile valley depth before tribological testing. ^4^ $\bar{{R_{v}}_{w}}$ is the average maximum profile valley depth after the Ball-on-Disc testing. ^5^ $\bar{{R_{dc}}_{0}}$ represents the average reduced depth of the roughness profile before tribological testing. ^6^ $\bar{{R_{dc}}_{w}}$ is the average reduced depth of the roughness profile after the Ball-on-Disc testing.

**Table 3:** Percentage variation (Δ*P*) of the profilometric surface parameters after the Ball-on-Disc tests for the investigated infill densities.

Infill [%]	Δ*P* ^1^ (*R_z_*) [%]	Δ*P* (*R_v_*) [%]	Δ*P* (*R_mr_*) [%]	Δ*P* (*R_dc_*) [%]
10	24.78	−7.79	−69.45	284.64
30	0.65	−21.71	−52.32	229.09
50	−19.10	−47.73	−80.82	242.55
70	15.19	−8.86	−32.89	176.62
90	−10.90	−39.73	−28.45	147.59
100	17.91	−18.81	−56.20	350.19

^1^ ΔP was calculated by using Equation (4).

**Table 4:** *STAI* values for the investigated infill densities.

Infill [%]	$\bar{{R_{a}}_{0}}$ ^1^ ± *SD* [µm]	$\bar{{R_{a}}_{w}}$ ^2^ ± *SD* [µm]	*STAI* ^3^ [%]
10	1.95 ± 0.12	3.99 ± 0.19	104.62
30	2.90 ± 0.49	4.26 ± 0.68	47.07
50	3.27 ± 1.08	4.04 ± 0.19	23.78
70	2.96 ± 1.18	4.23 ± 0.65	42.79
90	4.26 ± 0.23	5.05 ± 0.96	18.37
100	2.05 ± 0.60	4.36 ± 0.41	112.87

^1^ $\bar{{R_{a}}_{0}}$ represents the arithmetic mean roughness before tribological testing. ^2^ $\bar{{R_{a}}_{w}}$ is the average arithmetic mean roughness after the Ball-on-Disc testing. ^3^ *STAI* was calculated by using Equation (5).

**Table 5:** *PDI* values for the investigated infill densities.

Infill [%]	$\bar{{R_{p}}_{0}}$ ^1^ ± *SD* [µm]	$\bar{{R_{p}}_{w}}$ ^2^ ± *SD* [µm]	*PDI* ^3^ [%]
10	3.12 ± 0.38	9.06 ± 1.09	190.70
30	3.99 ± 0.79	8.78 ± 2.46	120.32
50	3.95 ± 0.95	9.72 ± 0.96	146.20
70	4.83 ± 1.60	10.41 ± 0.86	115.46
90	5.88 ± 0.36	13.03 ± 4.61	121.47
100	3.51 ± 0.64	10.71 ± 1.04	205.42

^1^ $\bar{{R_{p}}_{0}}$ represents the average maximum profile peak height before tribological testing. ^2^ $\bar{{R_{p}}_{w}}$ is the average maximum profile peak height after the Ball-on-Disc testing. ^3^ *PDI* was calculated by using Equation (6).

**Table 6:** *MRPI* values for the investigated infill densities.

Infill [%]	$\bar{{R_{mr}}_{0}}$ ^1^ ± *SD* [%]	$\bar{{R_{mr}}_{w}}$ ^2^ ± *SD* [%]	*MRPI* ^3^ [-]
10	2.68 ± 2.14	0.82 ± 0.67	0.31
30	2.56 ± 2.39	1.22 ± 1.34	0.48
50	4.20 ± 5.04	0.81 ± 0.29	0.19
70	1.47 ± 1.16	0.99 ± 0.62	0.67
90	1.11 ± 0.47	0.78 ± 0.34	0.72
100	2.16 ± 1.08	0.95 ± 0.47	0.44

^1^ $\bar{{R_{mr}}_{0}}$ represents the average material ratio before tribological testing. ^2^ $\bar{{R_{mr}}_{w}}$ is the average material ratio after the Ball-on-Disc testing. ^3^ *MRPI* was calculated by using Equation (7).

## Data Availability

The original contributions presented in this study are included in the article. Further inquiries can be directed to the corresponding author.

## Abbreviations

The following abbreviations are used in this manuscript:

AM	Additive Manufacturing
ANOVA	Analysis of Variance
ASA	Acrylonitrile Styrene Acrylate
ASTM	American Society for Testing and Materials
CF	Carbon Fibers
DIN	Deutsches Institut für Normung
FFF	Fused Filament Fabrication
IPA	Isopropyl Alcohol
ISO	International Organization for Standardization
MRPI	Material Ratio Preservation Index
PDI	Peak Deformation Index
PLA	Polylactic Acid
PLA-CF	Polylactic Acid reinforced with Carbon Fibers
STAI	Surface Texture Alteration Index
SD	Standard Deviation
WEI	Wear Efficiency Index

## Nomenclature

*A_net_*	Net Removed Area of Wear Track
*F*	Normal Load
*k*	Specific Wear Rate
*L*	Sliding Distance
*R*	Wear Track Radius
*R_a_*	Arithmetic Mean Roughness
*R_dc_*	Reduced Roughness Depth
*R_mr_*	Material Ratio
*R_p_*	Maximum Profile Peak Height
*R_q_*	Root Mean Square Roughness
*R_v_*	Maximum Profile Valley Depth
*R_z_*	Maximum Profile Height
*r*	Pearson Correlation Coefficient
*R* ^2^	Coefficient of Determination
*V*	Wear Volume
Δ*P*	Percentage Variation in Surface Parameter
*η* ^2^	Eta Squared (Effect Size)
